# Non-Faradaic Impedimetric Detection of Heavy Metal Ions via a Hybrid Nanoparticle-DNAzyme Biosensor

**DOI:** 10.3390/bios14070321

**Published:** 2024-06-27

**Authors:** Chrysi Panagopoulou, Evangelos Skotadis, Evangelos Aslanidis, Georgia Tzourmana, Annita Rapesi, Charalampos Tsioustas, Maria Kainourgiaki, Georgios Kleitsiotis, George Tsekenis, Dimitrios Tsoukalas

**Affiliations:** 1 Department of Applied Physics, National Technical University of Athens, 15780 Athens, Greece; chryssipanagopoulou@mail.ntua.gr (C.P.); or evaaslani@central.ntua.gr (E.A.); gtzourmana@mail.ntua.gr (G.T.); rapesisannita@yahoo.com (A.R.); charalampos_tsioustas@mail.ntua.gr (C.T.); mkainourgiaki@mail.ntua.gr (M.K.); gkleitsiotis@ieee.org (G.K.); dtsouk@central.ntua.gr (D.T.); 2 Department of Biomedical Engineering, The University of West Attica, 12243 Athens, Greece; 3 Microelectronics Research Group (MRG), Institute of Electronic Structure and Laser (IESL), Foundation of Research & Technology Hellas (FORTH), 70013 Heraklion, Greece; 4 Biomedical Research Foundation of the Academy of Athens, 11527 Athens, Greece; gtsekenis@bioacademy.gr

**Keywords:** heavy metal, biosensor, non-faradaic, impedance, EIS, DNAzymes, chromium, lead, nanoparticles, lab on chip

## Abstract

Due to rapid industrialization, novel water-quality monitoring techniques for the detection of highly toxic and hazardous heavy metal ions are essential. Herein, a hybrid noble nanoparticle/DNAzyme electrochemical biosensor is proposed for the simultaneous and label-free detection of Pb^2+^ and Cr^3+^ in aqueous solutions. The sensor is based on the combination of a two-dimensional naked-platinum nanoparticle film and DNAzymes, whose double-helix configuration disassembles into smaller fragments in the presence of target-specific heavy metal ions. The electrochemical behavior of the fabricated sensor was investigated with non-faradaic electrochemical impedance spectroscopy (EIS), resulting in the successful detection of Pb^2+^ and Cr^3+^ well below their maximum permitted levels in tap water. So far, there has been no report on the successful detection of heavy metal ions utilizing the non-faradaic electrochemical impedance spectroscopy technique based on advanced nanomaterials paired with DNAzymes. This is also one of the few reports on the successful detection of chromium (III) via a sensor incorporating DNAzymes.

## 1. Introduction

In the past decades, extensive industrialization has significantly impacted the environment globally, particularly in terms of the pollution of natural resources [1]. Industries such as pulp and paper, textile, cement, oil, leather, paint, and food, among others, normally produce large quantities of sludge and effluents [2], resulting in the generation of hazardous byproducts, such as heavy metals. As a consequence, such byproducts can easily transfer to air, water, and soil [3]. In particular, the contamination of aquatic systems is considered to be a major global issue due to the substantial volumes of wastewater produced by these industries [4].

Heavy metal ions pose a significant threat to water quality, as they are highly toxic, resistant to degradation, and prone to bioaccumulation and biomagnification within the food chain. The most commonly found heavy metals in waste water include chromium and lead, which cause risks for both human health and the environment [5]. The existence of such ions in aquatic ecosystems, even in trace amounts, can directly or indirectly impact living systems [6]. For instance, lead (Pb^2+^) is one of the most common and hazardous pollutants, and excess levels can lead to organ disorders, carcinogenicity, and genotoxicity [7]. Chromium is also characterized as a potentially toxic metal, mainly occurring in water in two oxidation states, Cr(III) and Cr(VI), primarily with the hexavalent compounds having toxic effects on humans, animals, plants, and microorganisms. In particular for human health, occupational exposure may damage the eyes, blood, respiratory and immune system [8]. In addition, Cr(III) is non-biodegradable, mutagenic, and genotoxic [9].

It is, therefore, of great significance to develop novel water-quality monitoring techniques, at both large and small scales, which enable selective detection of these water pollutants with sufficiently low limits [10]. Up to now, the most commonly used techniques to detect heavy metal ions in water include highly sensitive spectroscopic techniques like atomic absorption spectroscopy (AAS) [11], inductively coupled plasma optical emission spectrometry (ICP-OES) [12], inductively coupled plasma mass spectrometry (ICP-MS) [13], and atomic fluorescence spectroscopy (AFS) [14].

However, these approaches are frequently time-consuming, costly, demand advanced equipment and highly trained staff, and are not appropriate for on-site detection. In addition, these methods are only suitable for quantitative analysis and need to be coupled with other chromatographic techniques to conduct metal ion speciation. On the contrary, electrochemical techniques are more cost-effective, user-friendly, reliable, and applicable for in situ monitoring of contaminated samples [15]. Among existing electrochemical biosensing techniques, electrochemical impedance spectroscopy (EIS) is a well-established method to investigate properties of materials and electrode reactions. Since electrochemical processes form the foundation of numerous research fields, including energy conversion and storage, corrosion studies, as well as biosensors, this method is considered to be broadly applicable because it can provide deep insight into the electrochemical phenomena at an electrified interface. This is due to the fact that an electrochemical process occurring at the interface between an electrode and an electrolyte can be deconstructed into a series of intricate stages—such as mass transport, charge–transfer processes, and adsorption—in a single measurement. Based on the response of the electrochemical system, the use of transient techniques, such as EIS, facilitates the analysis of time-dependent mechanisms at specific frequencies [16].

EIS can function in two modes, namely faradaic and non-faradaic. The key distinction between the two lies in the use of a redox species in the faradaic mode. In faradaic EIS, the redox couple undergoes alternating oxidation and reduction via electron transfer to the metal electrode. Consequently, faradaic EIS necessitates the inclusion of a redox-active species and specific DC bias conditions to prevent depletion. It is worth noting that the redox species can damage biomolecules [17,18], resulting in the enhancement of biomolecular agglomeration, thus leading to less sensitive detection [19]. Conversely, non-faradaic EIS does not demand any extra reagent, allowing for real-time measurement and highly sensitive detection, and is therefore more suitable for point-of-care applications [20]. In addition, the non-faradaic mode of operation offers detection without the need of a reference electrode—which is required in faradaic mode—and hence, is amenable to miniaturization. The existing literature has a very limited number of research studies focused on HMI detection based on non-faradaic EIS biosensors. One study is an impedimetric sensor comprised of L-cysteine self-assembled on top of an interdigitated gold electrode for the detection of lead ions (Pb(II)) in tap water [21]. Another is an impedimetric sensor for the detection of Hg(II) in tap water, comprised of titanium dioxide (TiO_2_) microstructures deposited over interdigitated gold electrodes (Au-IDEs) [22]. However, these publications rely solely on capacitive changes at the electrode/electrolyte interface, while completely disregarding the resistive part of the impedimetric measurements, which furnishes crucial information about the entirety of the non-faradaic system. Additionally, the developed sensors were capable of successfully detecting only one type of HMI.

Emerging sensing technologies for HMI also utilize compact sensors and advanced nanomaterials along with specific biorecognition molecules, designed to create nanosensors with cost-effective fabrication, portability, and easy operation. Various nanomaterials, including gold nanoparticles [23], nanoporous alumina [24], and ZnO nanowires [25], have previously been employed as sensing layers to enhance the efficiency of non-faradaic impedimetric sensors. Functional biomaterials, including DNAzymes—enzymatic, single-stranded (ss), synthetic DNA sequences—are frequently employed in biosensing platforms for the highly sensitive and selective detection of heavy metal ions [26]. This is due to their ability to cleave and eventually break down when specific metal ions bind to designated sites within their DNA sequence. DNAzyme sensors for HMI detection operate with either fluorescent, colorimetric, or electrochemical signal readout as the sensing signal [27]. DNAzymes—when employed for HMI detection—are frequently paired with nanomaterials, with common applications involving the utilization of nanomaterials as optical quenchers [28], as optical enhancers due to surface plasmon resonance properties [29], quantum dots as signal reporters for chemiluminescence resonance energy transfer [30], or as suitable bases for DNA immobilization [31]. Nevertheless, to the authors’ knowledge, the detection of HMIs based on the combination of advanced nanomaterials and DNAzymes has not been previously recorded with the use of non-faradaic EIS.

In this study, a non-faradaic biosensor employing platinum (Pt) nanoparticles (NPs) and DNAzymes was developed for the simultaneous and label-free detection of HMI targets, namely lead (Pb^2+^) and chromium (Cr^3+^). The two-dimensional (2D) platinum (Pt) NP film is deposited in between interdigitated electrodes (IDEs) creating nano-gapped electrodes, on top of which target-selective DNAzymes are immobilized by employing thiol-functional groups. As previously reported by this group [32,33,34], DNAzymes are utilized not only because of their catalytic activity in the presence of HMIs, but also because they offer enhanced device conductivity by acting as inter-nanoparticle bridges and collapse selectively in the presence of the target substance. The sensor successfully detected concentrations of Pb^2+^ and Cr^3+^ that fell significantly below their permissible levels in tap water. Results discussed herein, expand our previously published work on the development of hybrid NP/DNAzymes biosensors for HMI detection [32,33,34] by comparing between biosensors employing either resistive or non-faradaic EIS measurements. It is worth noting that EIS is one of the most established techniques in the field of biochemical sensing, since it can provide extensive understanding of the interactions taking place at the devices’ electrode–electrolyte interface. Non-faradaic EIS biosensors significantly outperformed their resistive counterparts by achieving lower limits of detection and a higher overall yield. Furthermore, this is the sole report on the development of a non-faradaic impedimetric electrochemical biosensor functionalized with DNAzymes. In addition, the biosensor discussed herein is the only report on an impedimetric sensor (faradaic or non-faradaic) that has been further optimized by incorporating a 2D NP layer that serves a dual purpose: on the one hand the NP layer increases the number of available DNAzyme-binding sites on the biosensor’s surface (increased roughness, hence increased surface to volume ratio), while on the other, it enhances the biosensor’s impedimetric-response via the introduction of resistive charge–transport pathways through the NP layer. This constitutes a distinctive sensing mechanism [32,33,34] that is investigated via EIS for the first time in the current study.

All things considered, the proposed biosensor stands out as a promising device for cost-effective, highly sensitive and selective detection of multiple HMIs, as it provides rapid response, involves a straightforward fabrication process, and eliminates the need for the additional reference electrode required in faradaic EIS. The combination of double stranded (ds) DNA’s electrical properties [35] and the NP layer is a distinctive feature of a unique sensing scheme within the field of electrochemical biosensors. It is also noteworthy that, to the best of the authors’ knowledge, this is one of the few reports where the successful detection of Cr^3+^ using DNAzymes is discussed, regardless of the sensing technique. In order to evaluate the sensor’s specificity, different non-target HMIs were tested with the non-faradaic sensor, while real samples were also examined. Our current results render the developed biosensor appropriate for potential integration into portable and remote environmental monitoring systems, as well as water treatment and remediation platforms in the future.

## 2. Materials and Methods

The biosensors were fabricated on top of silicon substrates with a 300 nm thick thermal SiO_2_ oxide layer, following the typical procedure described in previous publications by this group [32,33,34,36]. In short, via conventional optical lithography, followed by e-gun metallization, gold interdigitated electrodes (IDEs) with a 10 μm electrode gap distance were patterned on top of the substrates, while a titanium layer (10 nm in thickness) was employed as an intermediate adhesion layer between the gold and SiO_2_, leading to a combined thickness of 40 nm for the IDEs after lift-off. Upon the completion of electrode fabrication, naked Pt NPs were deposited using DC magnetron sputtering, a physical vapor deposition technique that provides control over the conductivity/resistance of the fabricated devices [32]. The nanoparticles are synthesized at room temperature, showing good size dispersion in the range of 2–12 nm (mean diameter ~5 nm) [37]. The device’s resistance is monitored in situ and is mainly dependent on two factors: the size of the nanoparticles, which can be controlled by adjusting the distance of the platinum sputtering target and the deposition-substrate, and the NP surface coverage and density—affected by the deposition time. In this case, the required NP surface coverage is slightly below the percolation threshold, which is optimal for device sensitivity, aligning with previous findings reported by this group [32,33,34].

All reagents were purchased from Merck (Merch SA, Darmstadt, Germany), while all buffers were prepared utilizing deionized (DI) water obtained from a Millipore MilliQ system, possessing a resistivity of 18.2 MΩ cm at 25 °C. Oligonucleotides were purchased from Integrated DNA Technologies, BVBA (Leuven, Belgium) and their sequences for the detection of and Pb^2+^ and Cr^3+^ were, respectively, as follows: Gr5 catalytic strand: GTTCGCCATCTGAAGTAGCGCCGCCGTATAGTGACT and Ce13d catalytic strand: GTTCGCCATAGGTCAAAGGTGGGTGCGAGTTTTTACTCGTTATAGTGACT. DNA hybridization was achieved with a common substrate strand, whose sequence was AGTCACTATrAGGAAGATGGCGAAC. The catalytic strands contained a 5′ thiol C6 linker, with the aim of achieving immobilization on the active area of the sensors (meaning the IDEs surface area). The materials and reagents listed below were also employed for the surface’s functionalization and DNAzymes’ immobilization: phosphate buffered saline (PBS) of pH = 7.4; phosphate buffer 1 M, pH = 8, 0.001% tween20; MOPS buffer (3 (Nmorpholino)propanesulfonic acid) (50 mMMOPS/25 mM NaCl, pH = 7.5) and MES buffer (2-(N-morpholino)ethanesulfonic acid) (50 mM MES/25 mM NaCl/0.8 mM phosphate buffer, pH = 6); and 6-mercapto-1-hexanol (MCH).

Every preparation step was carried out at ambient temperature. The biochemical protocols used for surface functionalization and DNAzymes’ immobilization were identical to our previous publication of HMI detection via resistive biosensors [32]. To be more specific, thiol-modified DNA sequences have been employed, since it was concluded that the thiol-modified immobilization technique exhibited a better overall performance. In particular, the thiol-based devices showed higher sensitivity and also proved to be more cost-effective due to their simple and fast fabrication process.

**Figure 1:**
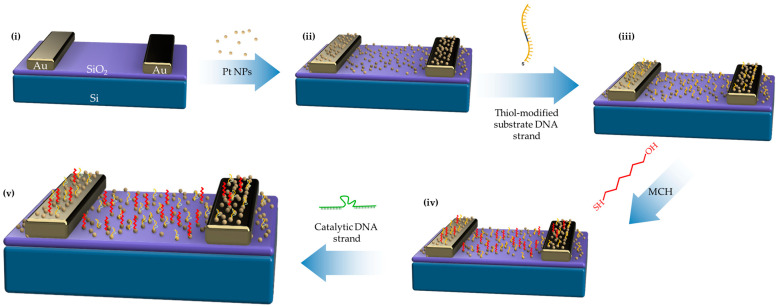
Schematic representation of the immobilization process for thiol-modified DNAzymes. (**i**) Si/SiO_2_ substrates have been patterned with Au interdigitated electrodes and have been used for the (**ii**) Pt NPs deposition step via the magnetron sputtering technique. (**iii**) The ssDNA substrate probes were immobilized on the sensors’ surface via drop-casting. MCH was employed (**iv**) in order to convey a blocking effect with a dual role; remove any non-specifically bound catalytic strands from the surface and act as an interaction barrier between single DNA strands. The final step of the process (**v**) involved the hybridization of the DNAzyme sequences with the immobilized substrate strands.

The immobilization process for thiol-modified DNAzymes can be seen in the schematic representation of Figure 1. In short, the ssDNA substrate probes had to be initially immobilized on the sensors’ surface via drop-casting on the IDEs. Next, MCH was employed in order to convey a blocking effect, so as to remove any non-specifically bound catalytic strands from the surface in order to act as an interaction barrier between single DNA strands. The final step of the process involved the hybridization of the DNAzyme sequences with the immobilized substrate strands. After the successful formation of the ds DNAzymes on the biosensor’s surface, the device could be immediately used for heavy metal ion detection and recognition while exposed to room temperature and humidity and without any special requirements. The device could also be stored in humid conditions at a temperature between 4 and 5 °C. Stored biosensors maintained their ability to successfully detect both heavy metal ion targets even after being stored in such conditions for more than a month.

**Figure 2:**
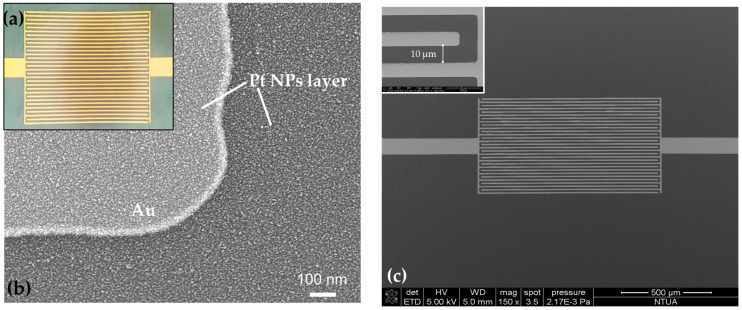
(**a**) Optical image of the interdigitated electrodes of a single sensor, with an inter-finger spacing of 10 μm. (**b**) SEM image of a single sensor on the margin of the gold electrode. Platinum nanoparticles having a mean diameter of 5 nm have been deposited via DC magnetron sputtering. (**c**) SEM overview image of a sensor prior to NP deposition. The inset shows the magnified picture of the IDEs, where the inter-finger spacing is equal to 10 μm.

**Figure 3:**
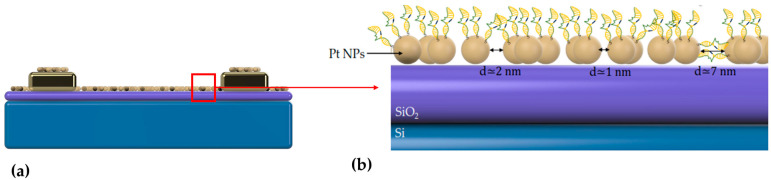
(**a**) Cross section (schematic) of the sensing device where the platinum (Pt) nanoparticle layer deposited on the electrodes (IDEs) can be seen. (**b**) Schematic representation of the thiol-modified DNAzyme functionalization distributed on top of the two-dimensional platinum (Pt) nanoparticle (NP) film. The Pt NP film offers a wide range of inter-nanoparticle gaps (noted as “d”) that can be under 1 nm and well over 2 nm.

The devices were characterized by optical microscopy measurements and field emission scanning electron microscopy (FE-SEM) (Figure 2) for every step of the immobilization process. The instrument used for the scanning electron microscopy was the Nova NanoSEM 230 (FEI Company, Hillsboro, Oregon) with a spot size of 3.0, accelerating voltage of 15 kV, tilting angle 0°, and working distance 5.4 mm. Optical microscopy displayed no notable variances between every biomolecular deposition/step, while SEM proved that electron charging on the sensors’ surface increased with each additional layer, which hindered further detailed SEM imaging. All DNA strands employed in this study, both substrate and enzymatic/catalytic, were marked with fluorescent tags, as detailed in [33], in order to verify every functionalization step via fluorescence microscopy. A more detailed schematic of the device’s hybrid DNAzyme–nanoparticle arrangement can be seen in Figure 3.

**Figure 4:**
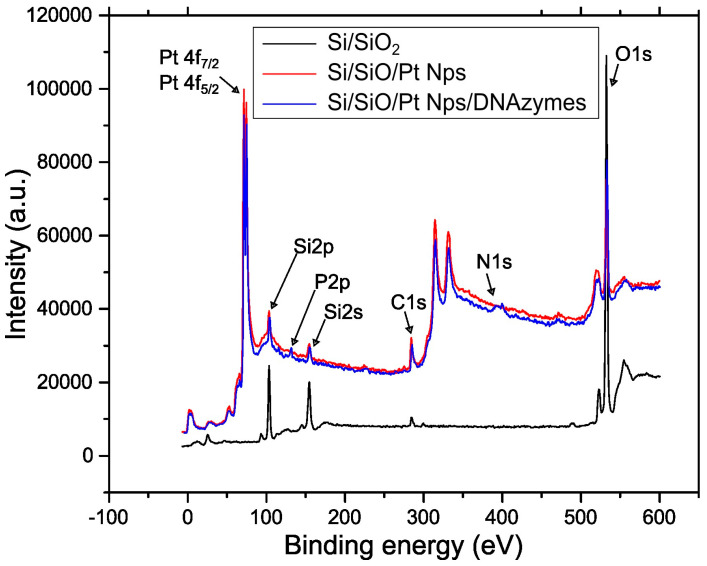
X-ray photoelectron spectroscopy (XPS) spectra results of a Si/SiO_2_ sample (black line), a Si/SiO_2_/Pt NPs sample (red line), and a Si/SiO_2_/Pt NPs sample modified with thiol DNAzymes (blue line).

In order to validate the immobilization of the DNAzymes layer on the sensor’s surface, X-ray photoelectron spectroscopy (XPS) analysis was employed. XPS analysis was performed as discussed in previous work by this group [32], with a MAX200 system. XPS spectra for a Si/SiO_2_ sample, a Si/SiO_2_/Pt NPs sample, and a Si/SiO_2_/Pt NPs sample modified with thiol DNAzymes, can be seen in Figure 4. From the XPS spectra, it is evident that the successful immobilization of the thiol-modified DNAzymes on top of the Pt NP layer results in P and N_2_ peaks that are characteristic of DNA presence. As is to be expected, P and N_2_ peaks did not occur in the case of the reference samples. Assuming, as a first approximation to the analysis of the data, that all the detected elements are uniformly distributed (both laterally and in depth) in the XPS analyzed volume of the surface region, we can then use the measured peak areas of characteristic peaks, one for each element, to obtain average atomic ratios between various elements normalized to 1 for Si in all samples. The peaks used in quantification were Si2s and Si2p, O1s, N1s, C1s, and Pt4f doublet. Appropriate relative sensitivity factors (RSF) were used from the database of the Surface Science Laboratory of the University of Patras, adjusted to the spectrometer operating conditions. The obtained quantification results under the assumption of spatial uniformity (average atomic ratios) for the analyzed samples are as follows: for the Si/SiO_2_/Pt NPs/DNAzymes sample: Si:O:N:Pt:P:C = 1:2.31:0.22:1.11:0.03:0.72, for the Si/SiO_2_/Pt NPs sample: Si:O:Pt = 1:1.74:1.45, and for the Si/SiO_2_ sample: Si:O = 1:2.00.

EIS measurements were conducted utilizing an Agilent 4284A precision LCR meter (Hewlett-Packard, Palo Alto, CA, USA) connected to the IDEs. The analyzer was connected to a Gateway G6-350 PC and controlled via LabVIEW Software (GPIB) (2011 version). All measurements were carried out within a custom-made electrochemical cell and recorded between a frequency range of 100 to 1,000,000 Hz with a modulation voltage of 50 mV. In order to verify that any change in the measured impedance could be solely attributed to target detection (HMI), 50 μL of buffer (suitable for each ion) was added on top of the IDEs (the active surface of the sensors) so that the system would reach an equilibrium state. This was further corroborated by the drop-casting of 5 μL of buffer solution before the addition of any HMI target, in order to ensure that the steady-state was not distorted. Increasing concentrations of HMIs dissolved in the appropriate analyte–MOPS buffer (for Pb^2+^) and MES buffer (for Cr^3+^) were drop-casted on top of the IDEs.

## 3. Results and Discussion

### 3.1. Detection of Heavy Metal Ions via EIS

EIS exhibits significant potential for the development of affordable, compact, and user-friendly portable devices for point-of-care applications, notably in the fields of medical diagnosis and environmental monitoring. This is due to the fact that it can relate the changes in electrical impedance to the reaction with the analyte of interest by producing an electrical signal proportional to the analyte concentration at the surface of the biosensing elements [38].

**Figure 5:**
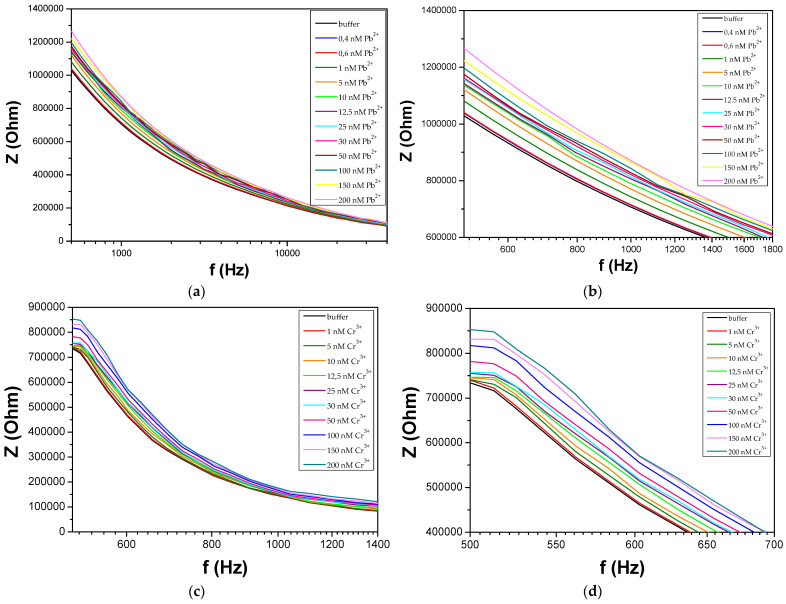
EIS responses of the devices at different concentrations of the two HMIs. The Bode plots of sensors functionalized with Pb-specific DNAzyme upon exposure to increasing concentrations of Pb^2+^ are presented for the entire frequency range in (**a**) and between 500 and 1800 Hz (**b**), while those functionalized with Cr-specific DNAzyme upon exposure to increasing concentrations of Cr^3+^ are presented for the entire frequency range in (**c**) and between 500 and 700 Hz in (**d**).

Since EIS, as a form of transfer function measurement, is frequently employed when examining linear time-invariant systems, the electrochemical impedance is a frequency dependent complex number, which can be expressed by the following formula:

(1) $$|Z|=\sqrt{{Z_{r}}^{2}+{Z_{i}}^{2}},$$

where $Z_{r}$ represents the real part and $Z_{i}$ signifies the imaginary part of impedance [16]. Given that the impedance is a complex value, the current can differ not only in terms of the amplitude, but it can also show a phase shift *φ* compared to the voltage–time function. Hence, one way of illustrating the results of an impedance measurement is by using a Bode plot, which plots log|Z| as a function of φ (or f) [39]. Herein, we demonstrate the Bode plots of total impedance and phase angle at a range of frequencies, as can be seen in Figure 5.

**Figure 6:**
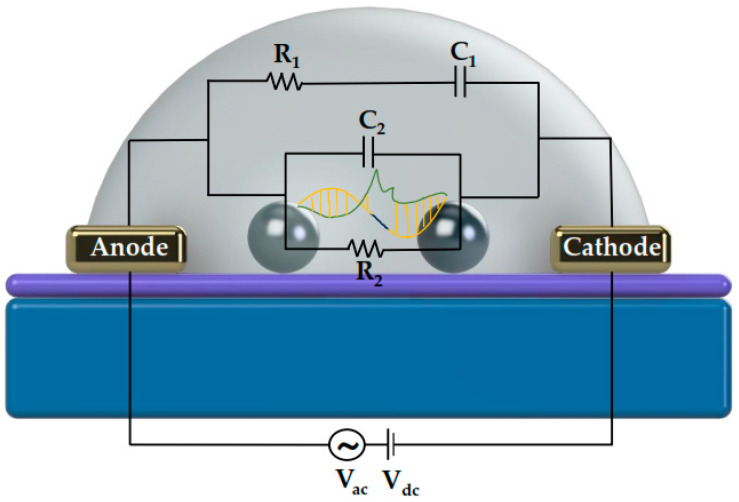
Equivalent circuit of the proposed device.

**Figure 7:**
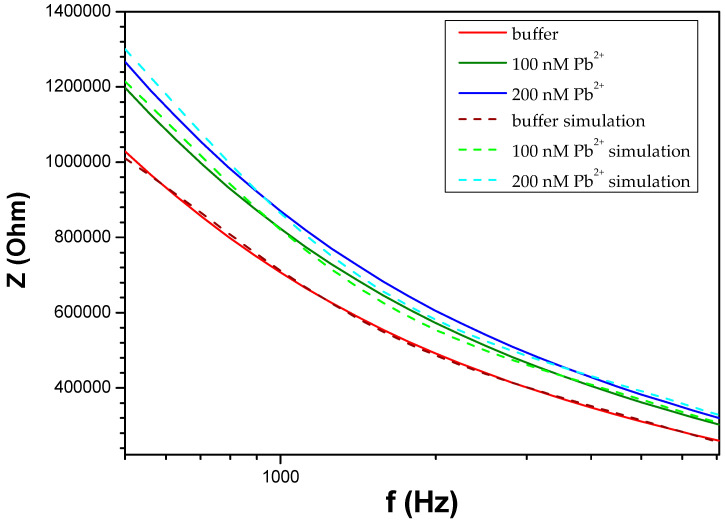
Fitting between experimental and the simulation data, according to the equivalent circuit model of Figure 3. Continuous and dashed lines represent experimental and simulated data, respectively.

In Figure 5, it is evident that exposure to the analyte leads to a substantial rise in the overall system impedance. In electrochemical impedance spectroscopy, where the electrolyte solution plays a crucial role in the system under investigation, usually four components are employed to characterize the impedimetric response: ohmic resistance, capacitance, constant-phase element, and Warburg impedance [39]. In a typical non-faradaic electrochemical system, the absence of a redox label excludes the parameters related to electron transfer and Warburg impedance that become infinite. Equivalent circuits utilizing ideal or distributed impedance elements arranged in series and/or in parallel, are usually employed in order to approximate the experimental impedance data. Our proposed equivalent circuit (Figure 6) consists of the ohmic resistance of the bulk electrolyte R_1_, which is in series with the dielectric capacitance of the solution C_1_ that depends on the permittivity of the solution (ε) and geometric characteristics of the nano-gapped electrodes in contact with the droplet (e.g., width and separation between the electrodes, droplet contact angle θ, etc.) [40]. These first two elements of the circuit are connected in series with the parallel combination of the double-layer capacitance C_2_ and the resistance R_2_ through the DNAzyme chains between the NP film. It is worth noting that in contrast to most publications related to non-faradaic or faradaic EIS, the model proposed in the current paper accounts for the existence of the NP layer; in fact, the presence of the Pt NP layer gives rise to the R_2_ component. Through numerical simulations (Figure 7), the parameters obtained from the compact model (Table 1) have proved to be self-consistent with the experimental data, confirming the validity and applicability of this model for similar biosensing systems (i.e., NP-modified biosensors and non-faradaic EIS measurements). To be more precise, in order to compute the passive elements $R_{i}$ and $C_{i}$ we first calculated the total resistance of the equivalent circuit of Figure 4, as shown in (2):

(2) $$Z_{total}=\frac{j\omega \left(R_{1}R_{2}C_{1}\right)+R_{2}}{{\left(j\omega \right)}^{2}\left(R_{1}R_{2}C_{1}C_{2}\right)+j\omega \left(R_{1}C_{1}+R_{2}C_{2}+C_{1}R_{2}\right)+1}$$

Using MATLAB’s optimization toolkit (version R2019a), specifically the fminsearch function, an unconstrained nonlinear minimization solver, we defined the objective function shown in (3) to minimize it:

(3) $$Objective\;Function={\left|\log\left({mag}_{data}\left(\omega \right)\right)-\log\left({mag}_{sim}\left(\omega \right)\right)\right|}^{2}$$

Here, ${mag}_{data}$ represents the experimental data, while ${mag}_{sim}$ is the magnitude response (computed using the bode function) of the total impedance at the corresponding frequencies of the experimental data.

At frequencies above 10 kHz, the impedance is solely attributed to the constant electrolyte resistance, regardless of any surface alterations or the presence of the targeted analyte. This corresponds to the flat, horizontal line observed in all of the Bode plots. At lower frequencies (*f* < 10^2^ HZ), the capacitive characteristics of the system prevail. At intermediate frequencies, both capacitance and resistance impact the impedance [40]. In contrast to the majority of published work based on impedimetric detection, herein changes in the as-measured conductance are not due to a changing charge–transfer resistance to the metal electrode, but can be understood in the context of counter-ion conduction on the DNA backbone, as well as due to the “conductive” bridging of distinctive NPs or NP clusters by hybridized DNAzymes [41]. This is why we report the impedance percentage difference before and after exposure to the analyte at 500 Hz; it is in this intermediate frequency that the largest changes in impedance have been recorded. As a consequence, the significant increase in total device impedance in the presence of the targeted analyte can be attributed to two phenomena happening simultaneously, one concerning the R component, and the other the C part. It is also worth noting that the increased sensitivity in the 500 Hz regime is also certified by the simulation data, as extracted by the proposed equivalent-circuit model.

**Figure 8:**
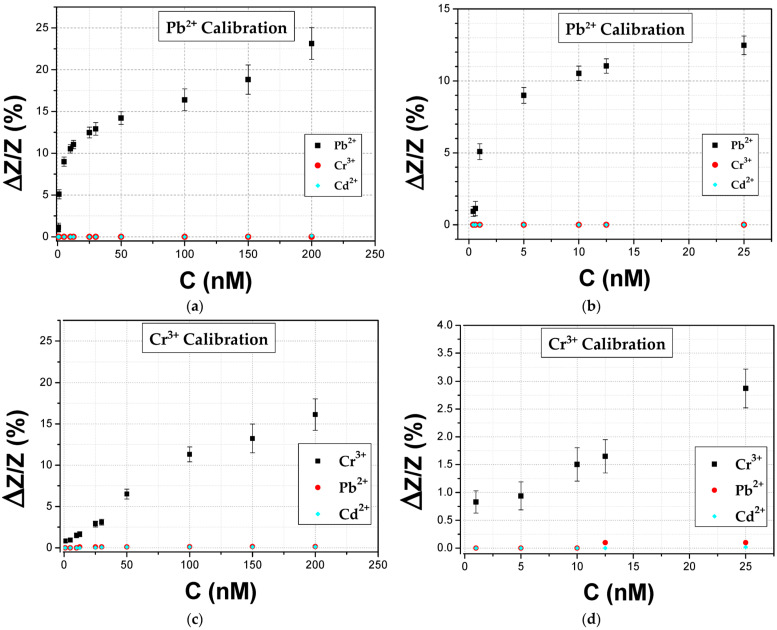
Impedimetric biosensor calibration-curves obtained at 500 Hz for the two HMIS: (**a**) Pb^2+^ and (**c**) Cr^3+^. Enlarged graphs for these curves are presented in (**b**,**d**) for the two metal ions, respectively. Biosensor response and control experiments are represented by black squares and red closed discs for either Pb^2+^ or Cr^3+^ and cyan diamonds for Cd^2+^.

In Figure 8, the response of the proposed biosensors towards Pb^2+^ and Cr^3+^ for thiol-modified catalytic strands can be seen. The mean base resistance or R_0_ (R_0_: initial device resistance) of the sensors used in these experiments varied between 700 and 2000 kΩ with a standard deviation of 7.3%. For the detection of each distinctive HMI concentration, 10 different DNAzyme biosensors were used in total in order to calibrate the biosensors, while the standard deviation of these precision measurements was between 0.25% and 1.9%. The results correspond to a relative change in impedance (ΔΖ/Ζ%) at a frequency equal to 500 Hz; in particular, there was an increase in the measured impedance of the device as shown in the Bode plots of Figure 5, as a result of the cleavage of the substrate strand of the DNAzymes. The sensors had a limit of detection (LoD) of 0.4 nM and 1 nM for Pb^2+^ and Cr^3+^, respectively. For Cr^3+^, a linear response range was obtained from 5 to 200 nM, while for Pb^2+^ two distinctive linear regions can be established: the first one from 400 pM to 10 nM and the second one from 50 nM to 200 nM. The cross-sensitivity and selectivity of the biosensors was also tested during dedicated control experiments, by adding a buffer solution containing a non-specific HMI to the respective DNAzyme-functionalized biosensor before the introduction of the target-specific HMI, as can be also seen in Figure 8.

**Figure 9:**
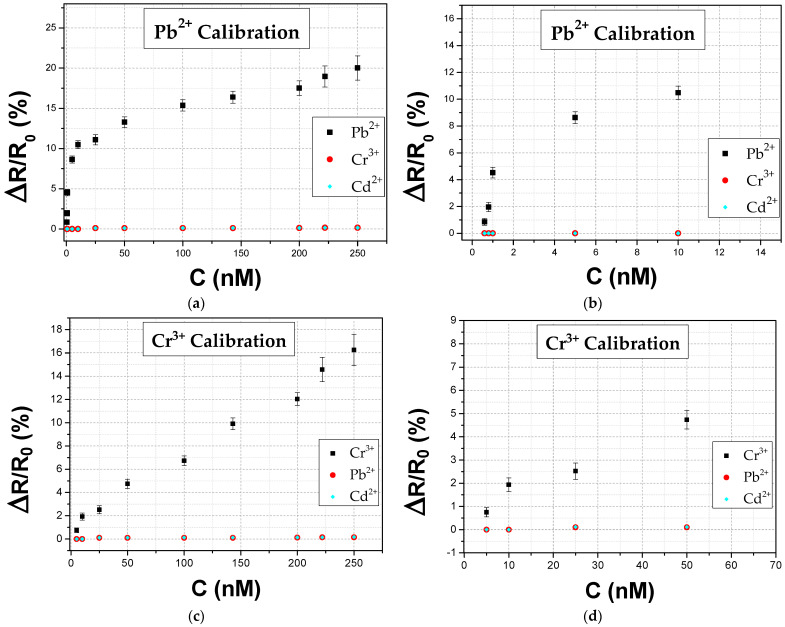
Resistive biosensor calibration curves obtained at 500 Hz for the two HMIS: (**a**) Pb^2+^ and (**c**) Cr^3+^. Enlarged graphs for these curves are presented in (**b**,**d**) for the two metal ions, respectively. Biosensor response and control experiments are represented by black squares and red closed discs for either Pb^2+^ or Cr^3+^, respectively, and cyan diamonds for Cd^2+^.

As to be expected, the results are improved compared to our previous work on heavy metal ion detection with the resistive biosensing technology [32], due to the contribution of the C component in device performance. In order to compare the two measurement techniques (i.e., non-faradaic EIS and resistive measurements) an additional experimental set has been performed for resistive biosensors, under the same unified conditions. The resistive biosensors were measured by following the characterization process described at length in [32,34]; the sensors’ resistance was monitored in situ by a Keithley 2400 Multimeter under a 1 V DC bias. Every sample was positioned on top of a printed circuit board (PCB) with the ability to measure up to eight sensors simultaneously in a homemade electrochemical cell, thanks to a custom-made multiplexer switch board which was connected to the Keithley instrument. The overall system was controlled via a custom-made LabVIEW application with the dynamic response of the biosensors appearing on the PC screen. The primary steps of measurement were the same as in the EIS setup, meaning that an initial stabilizing amount of buffer (suitable for each ion) was added on top of the IDEs before any HMI addition; the HMI detection was reported as an increase in the sensor’s resistance. The sensors’ response was similar to the one previously presented in [32], as can be seen in Figure 9.

The results show the relative increase in resistance (ΔR/R_0_%), corresponding to the dissociation of the substrate DNAzyme into two smaller fragments when exposed to target HMI. The resistive sensors had a limit of detection (LOD) of 0.8 nM and 10 nM for Pb^2+^ and Cr^3+^, respectively, which is considerably higher compared to the LOD achieved via non-faradaic detection. Apart from outperforming the resistive biosensors in terms of sensitivity, the impedimetric biosensors also exhibited higher yield; 71% of fabricated impedimetric sensors were measured successfully compared to 42% of resistive biosensors. It is also worth noting that initial device resistance (R_0_) in the case of resistive biosensors has to be in the range of 500–950 kΩ for the successful operation of the device. Device resistance is connected to NP deposition time, hence NP surface coverage; in the case of resistive biosensors, specific NP surface coverage is necessary for successful inter-nanoparticle bridging, hence device operation. In contrast, the impedimetric sensors feature a wider R_0_ range that is between 700 kΩ and 2000 kΩ; as a consequence, there is no need for in situ monitoring of device, hence the NP fabrication process is accelerated, resulting in significantly faster and easy fabrication. The precision measurements had comparable standard deviation values; the value for the resistive biosensing technology was reported in the range of 0.2% to 1.5%. The linearity of the response calibration curves is also similar for both measurement methods, while the control experiments proved that both types of biosensors are characterized by good cross-sensitivity and selectivity. The one aspect lacking in the impedimetric detection method is the more complex measurement setup and data analysis required to acquire information; on the contrary, resistive biosensing technology relies on simpler instrumentation and instant experimental results (response time between 7 and 18 s), which do not demand any further data processing. A comparison between the two distinct measurement regimes, namely non-faradaic EIS and resistance, is showcased as Table 2.

**Figure 10:**
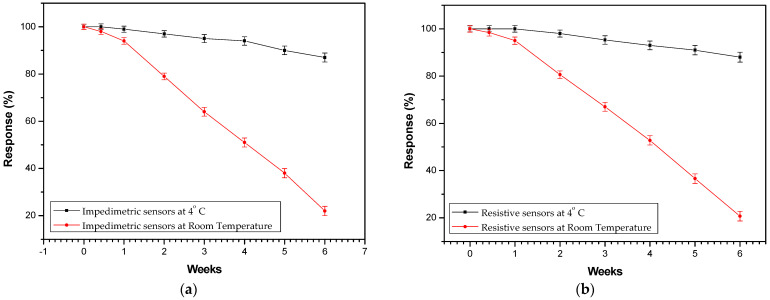
Effect of storage conditions on the performance of the (**a**) impedimetric and (**b**) resistive biosensors. Two storage conditions were assessed: one at 4 °C and the other at room temperature, over a period of up to 6 weeks post-fabrication. The vertical axis displays the reduced signal from the sensors’ responses, which is normalized to the initial response measured immediately after fabrication in “week 0” for 5 nM of Pb^2+^. The error bars indicate the standard deviation derived from measurements of five distinct sensors at each time interval.

Storage stability data for both types of biosensors were also collected (Figure 10), in accordance with previously reported results of our group [34]. By storing the biosensors in a temperature of 4 °C, stability could be achieved over a period of 6 weeks. On the contrary, sensors stored at room temperature proved to have deteriorated sensing abilities over time, losing their overall sensitivity in 4 weeks. Three days was calculated to be the maximum time capacity a sensor could be stored at room temperature without showing any performance deterioration. It is also evident from the graph that both impedimetric and resistive sensors exhibit almost identical storage abilities since they are based on the same fabrication scheme.

Finally, the proposed biosensors exhibit prospects of reusability when following a specific process, described at length in [34]; the devices need to be washed with the respective buffer solution before the remaining immobilized DNA strands, previously fractured by the HMI addition, can once again be hybridized according to the steps of Section 2. However, during the DNAzymes’ cleavage, small fragments of the substrate strands remain attached to the surface; since the complete removal of those fragments is required, a process which involves breaking the covalent bond on the sensor’s surface, the overall regeneration of the biosensing system is not cost-effective [32].

### 3.2. Heavy Metal Ion Detection in Real Samples

**Figure 11:**
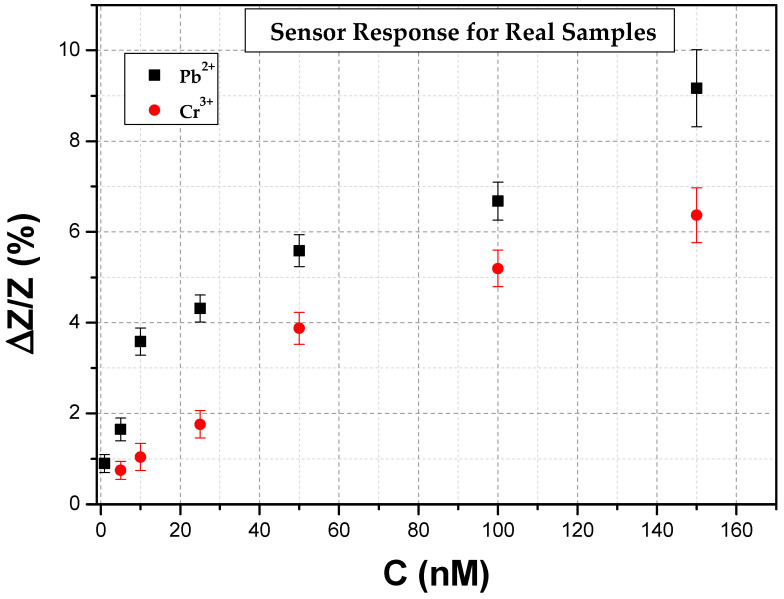
The sensors’ response obtained at 500 Hz for Pb^2+^ and Cr^3+^ impedimetric detection in real samples.

**Figure 12:**
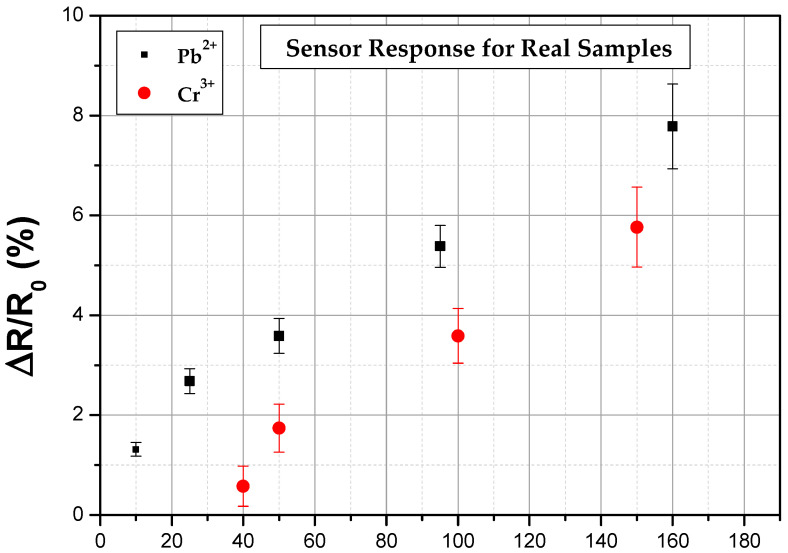
The sensors’ response obtained for Pb^2+^ and Cr^3+^ resistive detection in real samples.

In order to validate the feasibility of the method to identify heavy metal ions in aqueous solutions, we enhanced the scope of our research compared to our previous work [32] by assessing the performance of the biosensing platform with real samples, namely tap water. Control experiments were conducted by adding known concentrations of the two types of HMIs (with a sample size of five sensors for each HMI concentration). A linear response of Pb^2+^ and Cr^3+^ detection with an LOD as low as 1 nM and 5 nM, respectively, was achieved (Figure 11). As can be seen, the linear response of the sensors was slightly distorted, especially in the case of Pb^2+^. Control experiments in real samples were also conducted with the use of resistive biosensing. Expanding on our previous work based on such sensors [32], the detection of Pb^2+^ and Cr^3+^ was also achieved (Figure 12), however, with lower LOD compared to the impedimetric sensors, namely 10 nM and 40 nM, respectively. The sensitivity of the impedimetric detection method is evidently higher, further highlighting the fact that integrating the capacitance value via the non-faradaic EIS method results in more sensitive and reliable biosensors.

The sensitivity was, in both cases, lower compared to devices measuring heavy metal ions in buffer solutions. This can be attributed to the fact that the ionic nature of buffer solutions affects biological processes, including enzyme activities—in our case, the DNAzymes’ cleavage. Generally, biological systems operate in conditions where ion specificity modulates biomacromolecule interactions [42]. Thus, the DNAzymes’ configuration and interactions are facilitated under the appropriate buffer environments. Hence, the plain aqueous solutions of real samples can reduce, to some extent, the sensitivity of the biosensors. However, the results exhibit potential for the future integration of these non-faradaic biosensors for point-of-care applications, such as microfluidic channels that consist of different target-specific DNAzyme devices, since the biosensors are capable of detecting the respective HMIs in concentrations well below their maximum permitted levels.

## 4. Discussion

The mechanism of charge–transport in nanoparticle films deposited via sputtering, with a surface coverage slightly below the percolation threshold, has been extensively addressed in prior publications by this research group [32,33]. In brief, charge–transport is dominated by quantum mechanical phenomena like tunneling and/or variable range hopping, while devices operating within this range usually feature a thermally activated Arrhenius-type conductivity mechanism [34].

On the other hand, the electronic orbital overlap of the DNA bases as well as the possibility to control DNA sequencing and length (in vastly possible combinations), renders DNA a highly effective one-dimensional system for charge–transport [43,44]. It has been suggested that aqueous environments contribute to enhanced DNA conductivity due to better base-pair stacking and coupling as well as more stable helix conformation compared to dry conditions. It has also been proven that ssDNA is more conductive than that of corresponding dsDNA [42,45], while guanine–cytosine base pairs have been found to display higher conductance compared to others that act as electric barriers [35].

**Figure 13:**
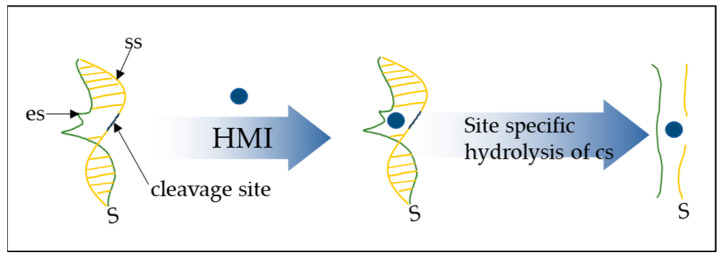
The DNAzymes consist of the enzyme strand (es), the substrate strand (ss), and the cleavage site; upon recognition of a heavy metal ion (HMI) target, the substrate strand is cleaved [32].

In previously published results by this group [32,33,34], hybrid, resistive nanoparticle/DNAzyme-based biosensors have been thoroughly investigated; herein ds DNAzyme dissociation was investigated via non-faradaic EIS, however, the explanation for the resistance component of the impedance is still applicable. In particular, the presence of hybridized DNA chains within the NP film can ultimately alter the sensor’s resistance/conductivity. This is supported by the fact that dsDNA functions as a conductive link between distinctive nanoparticles or NP aggregates, thus creating new conductive pathways for charge transport. As a result, DNAzymes utilize metal ions to carry out catalysis [26] (Figure 13), thus leading to the fracture of the conductive bridges between the NPs and a significant increase in resistance [32,33,34]. Previously published results by this group have indicated that thiol-modified DNAzymes are designed in order to bind directly to Pt NPs [46], otherwise the DNA molecules cannot be attached to the sensor’s surface.

On the other hand, the present experimental results indicate that the capacitance value is decreasing with increasing target concentration. This capacitance variation is in accordance with results observed in the respective literature [47,48,49,50], where non-faradaic capacitive biosensors were developed. To be more specific, the decrease in capacitance in this work can be attributed to the accumulation of biomolecules within the electrical double layer (EDL) [51]. As already discussed herein, and in contrast to prior research concerning impedimetric DNA detection, the design of our proposed biosensor employs an additional 2D network of nanoparticles and DNA chains; while self-assembled ssDNA-layers in most reported impedimetric biosensors usually serve as an insulator in conjunction with the electrical double layer [48], in our case, the DNAzyme layer is hybridized prior to any exposure to the target analytes. DNA-functionalized electrodes generally cause higher non-faradaic electric transient current than bare electrodes, which corresponds to higher effective salt concentration in the region near the metal electrode [52]. Upon exposure to target HMI, the substrate strands are cleaved, leading to their charged fragments accumulating in the EDL and causing charge perturbation, which results in the system’s overall interface capacitance decrease.

In a typical non-faradaic electrochemical system, where the absence of a redox label excludes the parameters related to electron transfer and Warburg impedance, the imaginary part of impedance is inversely proportional to the electrical double-layer capacitance [49], while the resistance value is proportional to the $Z_{r}$ component of the system’s impedance. In alignment with established research in the field of non-faradaic EIS where in-solution DNA hybridization has been investigated [40,53,54,55], we reported the expected opposite results, hence an overall rise of impedance due to the DNAzymes’ cleavage upon exposure to target HMI. Building upon our previous articles on heavy metal ion detection based on resistance measurements [32,33,34], this work allowed for deeper understanding of the electrochemical processes via the introduction of EIS characterization leading to an overall improved biosensor performance. This is reflected on the increased sensitivity offered by the proposed device, for laboratory and real samples alike, as well as the overall yield and reliability which were highly improved compared to resistive biosensors, owing to the existence of the C component. The sensing mechanism for resistive biosensors relies solely on the collapse of the DNAzyme inter-nanoparticle bridging (resistive component); in contrast, impedimetric biosensors utilize both resistive and capacitive components, recorded as an overall rise in impedance. All of the above are validated through the experimental measurements as well as from the successful fitting of the equivalent circuit model.

## 5. Conclusions

A novel electrochemical biosensor has been developed for the detection of two distinct heavy metal ions, namely lead (Pb^2+^) and chromium (Cr^3+^). The biosensor relies on a combination of noble metallic nanoparticles (i.e., platinum) and DNAzymes, attached on the nanoparticle film through a thiol anchoring group. The device demonstrated the capability to detect both heavy metal ions at concentrations well below their permissible levels in tap water. Expanding on our previous publications related to the detection of heavy metal ions using resistive biosensors, this study has enabled a more profound comprehension of the device’s electrochemical interactions by incorporating the system’s capacitance via non-faradaic EIS measurements, while also conducting comparative experiments based on the two different biosensing detection methods under the same unified conditions.

Table 3 presents a list of reported sensors using various sensing principles for HMI detection schemes, categorized into general and most commonly used techniques, where DNAzymes were utilized and biosensors measured via non-faradaic EIS. To the authors’ knowledge, the proposed biosensor is the sole DNAzyme-based biosensor for the detection of heavy metal ions that was measured with the use of non-faradaic electrochemical impedance spectroscopy. As a result, it allows for real-time, highly sensitive and label-free detection since it does not require the use of any redox species or a reference electrode (like in faradaic measurements). It is also worth noting that the developed biosensor is characterized by a unique impedimetric sensing mechanism, whether faradaic or non-faradaic, through the integration of a 2D nanoparticle layer serving a dual role, i.e., acting as expanded nano-gapped electrodes and as additional binding sites for DNAzyme immobilization.

In summary, the suggested biosensor emerges as a promising device for the cost-effective, label-free, sensitive, and selective detection of various heavy metal ions. This is also highlighted by its short response time, low power consumption, simple fabrication process, and experimental set-up. Future work entails the development of a multi-sensing array capable of simultaneously detecting and screening additional heavy metal ions (HMIs); this could also encompass a broader range of environmental contaminants. The ultimate objective of this study is to incorporate these biosensors into a single, disposable, and cost-effective platform (e.g., a lab-on-chip system), which would be able to facilitate the multiplexed detection of both—or potentially more—HMIs in a single measurement. Effortless automation makes it particularly suitable for remote and autonomous environmental monitoring systems or water treatment systems, as we move further into the era of the Internet of Things (IoT). Future research efforts will also involve further sensor optimization for higher sensitivity of the device, hence leading to even lower limits of detection (LoD), in particular for real samples, as well as developing appropriate strategies for sensor reusability.

## Figures and Tables

**Table 1:** Parameters obtained from the equivalent circuit-model of the biosensor.

Pb^2+^ Addition	R_1_ (Ohm)	C_1_ (10^−10^ Farad)	R_2_ (Ohm)	C_2_ (10^−11^ Farad)	R^2^ Value
0 (buffer)	819,714.289	1.058	1,332,126.638	7.048	0.960
100 nM	819,714.289	1.058	1,719,696.480	5.567	0.942
200 nM	819,714.289	1.058	1,903,953.434	5.080	0.934

**Table 2:** Comparison between the two different measurement regimes of the reported biosensors, namely non-faradaic impedimetric and resistive sensors.

Properties	Non-Faradaic EIS	Resistance Measurements
Analytes	Pb^2+^ and Cr^3+^	
Fabrication/reagents/materials	Same for both detection schemes	
Limit of detection (LOD)	0.4 nM for Pb^2+^ and 1 nM for Cr^3+^	0.8 nM for Pb^2+^ and 10 nM for Cr^3+^
Standard deviation values	0.4–1.9%	0.2–1.5%
Yield	71%	42%
Linearity	Similar	
Cross-sensitivity and selectivity	Similar	
Measurement setup and data-analysis	Complex	Simple
Limit of detection (LOD) for real samples	1 nM for Pb^2+^ and 5 nM for Cr^3+^	10 nM for Pb^2+^ and 40 nM for Cr^3+^

**Table 3:** List of various reported sensors for HMI detection.

Metal Ion/Ions	Detection Technique	Limit of Detection	References
***General detection techniques***			
Cu^2+^, Fe^3+^, Ni^2+^ and Zn^2+^	Atomic absorption spectrometry (AAS)	41, 61, 63, and 12 μg/kg	Trindade et al. [11]
Al^3+^, Ca^2+^, Cd^2+^, Co^2+^, Cu^2+^, Fe^2+^, Fe^3+^, Mg^2+^, Ni^2+^, Pb^2+^, and Sr^2+^	Inductively coupled plasma optical emission spectrometry (ICP-OES)	0.03–0.44 μg/L	Losev et al. [12]
Trace Cu, Zn, Cd, Hg, Pb, and Bi	Inductively coupled plasma mass spectrometry (ICP-MS)	49, 43, 4.2, 6.1, 13, and 18 ng/L	Wang et al. [13]
Pb^2+^	Atomic fluorescence spectroscopy	0.004 μg/L	Beltrán et al. [14]
***Techniques where DNAzymes were used***			
Hg^2+^, Ni^2+^, and Ag^+^	DNAzymes as optical quenchers	0.11 nM, 7.8 μM, and 0.25 nM	Pavadai et al. [28]
Hg^2+^	DNAzymes for colorimetric detection	10 pM	Chen et al. [29]
Hg^2+^	DNAzymes and quantum dots for chemiluminescent and chemiluminescence resonance energy transfer	10 nM	Freeman et al. [30]
Cu^2+^ and Hg^2+^	DNAzyme-functionalized single-walled carbon nanotubes for electrochemical impedance detection	0.01 and 5 nM	Wang et al. [31]
Pb^2+^, Cd^2+^, and Cr^3+^	DNAzymes based on platinum nanoparticles for resistive detection	0.8 nM, 1 nm, and 10 nM	Skotadis et al. [32]
***Non-faradaic electrochemical impedance spectroscopy (EIS)***			
Pb^2+^	L-cysteine on Au-IDE for non-faradaic EIS	45 pM	Assaifan et al. [21]
Hg^2+^	TiO_2_ microstructures on Au-IDEs for non-faradaic EIS	60 pM	Assaifan et al. [22]
*Pb^2+^ and Cr^3+^*	*DNAzymes immobilized on Pt nanoparticles for non-faradaic EIS*	*0.4 nM and 1 nM*	*This work*

## Data Availability

Data supporting reported results can be found here: 10.6084/m9.figshare.25288213.
